# Treatment with bexarotene, a compound that increases apolipoprotein-E, provides no cognitive benefit in mutant *APP*/*PS1* mice

**DOI:** 10.1186/1750-1326-8-18

**Published:** 2013-06-13

**Authors:** Katherine D LaClair, Kebreten F Manaye, Dexter L Lee, Joanne S Allard, Alena V Savonenko, Juan C Troncoso, Philip C Wong

**Affiliations:** 1 Department of Pathology, The Johns Hopkins University School of Medicine, 720 Rutland Avenue, Ross 558, Baltimore, MD 21205, USA; 2 Department of Neuroscience, The Johns Hopkins University School of Medicine, 720 Rutland Avenue, Ross 558, Baltimore, MD 21205, USA; 3 Department of Neurology, The Johns Hopkins University School of Medicine, 720 Rutland Avenue, Ross 558, Baltimore, MD 21205, USA; 4 Program in Cellular and Molecular Medicine, The Johns Hopkins University School of Medicine, 720 Rutland Avenue, Ross 558, Baltimore, MD 21205, USA; 5 Department of Physiology and Biophysics, Howard University College of Medicine, 520 W Street, NW Suite 2305, Washington, DC 20059, USA

**Keywords:** Bexarotene, Alzheimer’s disease, Mouse model, RXR agonist, APOE, Cognition

## Abstract

**Background:**

Though the precise cause(s) of Alzheimer’s disease (AD) remain unknown, there is strong evidence that decreased clearance of β-amyloid (Aβ) from the brain can contribute to the disease. Therapeutic strategies to promote natural Aβ clearance mechanisms, such as the protein apolipoprotein-E (APOE), hold promise for the treatment of AD. The amount of APOE in the brain is regulated by nuclear receptors including retinoid X receptors (RXRs). Drugs that activate RXRs, including bexarotene, can increase APOE and ABCA1 production, and have been shown to decrease the Aβ burden and improve cognition in mouse models of Aβ amyloidosis. Although recent bexarotene studies failed to replicate the rapid clearance of Aβ from brains, behavioral and cognitive effects of this compound remain controversial.

**Findings:**

In efforts to clarify these behavioral findings, mutant *APP*/*PS1* mice were acutely dosed with bexarotene. While ABCA1 was upregulated in mutant *APP*/*PS1* mice treated with bexarotene, this drug failed to attenuate Aβ plaques or cognitive deficits in these mice.

**Conclusions:**

We recommend rigorous preclinical study to evaluate the mechanism and utility of such a compound for AD therapy.

## Introduction

The principle pathological characteristics of Alzheimer’s disease (AD) include the extracellular deposition of β-amyloid (Aβ) plaques and intracellular aggregation of tau in the brain, abnormal synaptic function, and chronic inflammatory responses in neural tissue. A well-studied mouse model of Aβ amyloidosis is the double transgenic *APP*_*swe*_/*PS1*_*ΔE9*_ mouse that exhibits Aβ plaques in the hippocampus and cortex beginning at 6 months of age [1]. The progression of Aβ deposition occurs more rapidly in these transgenic females than in males [2]. While Aβ has a clear link to AD – either directly or through the processing mechanisms of its precursor protein APP – Aβ is also present in healthy individuals in the form of low molecular weight soluble Aβ peptides that likely serve physiological roles [3]. Levels of Aβ peptides are managed by clearance mechanisms in the brain that activate microglia to facilitate the removal of Aβ from the extracellular space [4]. The most prominent of these clearance components is the cholesterol transporter apolipoprotein-E (ApoE) because the *E4* allele as compared to that of *E2* or *E3* is the most significant risk factor for the development of non-familial (sporadic) AD. Individuals with *APOE4* have a pronounced increase in Aβ oligomerization [5] and AD incidence [6,7] relative to carriers of the other APOE alleles. *APOE4* has also been found to have decreased affinity for Aβ compared to *APOE3* or *APOE2*, but only when activated by lipidation [8], which implies that dysfunction in the regulation and action of APOE isoforms can play contributing roles in the pathogenesis of AD. The convergence of this data indicates that promoting APOE may be a productive therapeutic strategy in AD.

The transcription of *APOE* is facilitated by activation and heterodimerization of the nuclear receptor retinoid X receptor (RXR) with its partner receptors peroxisome activated receptors (PPARγ) or liver X receptors (LXR). These nuclear receptors also activate lipidators such as ABCA1 [9,10], which appear necessary for the Aβ clearing abilities of ApoE [8,11,12]. Therefore, a number of RXR agonists have been tested as a potential therapeutic mechanism of increasing Aβ clearance through ApoE. Bexarotene is one such agonist, which is selective for RXR and is FDA-approved for the treatment of cutaneous T-cell lymphoma. Cramer *et al*. [13] conducted studies with bexarotene in transgenic mouse models of Aβ amyloidosis. They reported that acute treatment with bexarotene upregulated ABCA1 and ApoE, rapidly reduced the Aβ plaque burden in the brain, and ameliorated cognitive deficits in these models. However, several groups have failed to replicate the effect of bexarotene on Aβ plaque burden in these and other related mouse models, despite achieving upregulation of the proposed targets ApoE and ABCA1 [14-17]. While the original experiments were conducted on mouse models (*Tg2576* and *APP*_*swe*_/*PS1*_*ΔE9*_) that exhibit significant gender-related pathological differences (*18 and 2*, *respectively*), no distinction of gender was made in their analysis [13]. In using a small cohort of mice in the studies, these gender differences could skew treatment groups leading to a false positive treatment effect. Moreover, gender-related differences are important in the evaluation of any RXR agonist or other compounds influencing this pathway because ApoE function can be modified by gender [19,20].

In this study, we attempted to clarify this gender discrepancy by measuring brain Aβ plaque load in male and female cohorts of *APP*_*swe*_/*PS1*_*ΔE9*_ mice acutely dosed with bexarotene. We used two different formulations of bexarotene, one in DMSO (as used by Cramer *et al*. [13]) and one in corn oil to assess any potential issues with DMSO toxicity. Since RXR and LXR agonists have been shown to modulate microglial activity to dampen their inflammatory responses and enhance their clearance abilities [21], we also examined microglial activation in these treated mice. Finally, no study has yet replicated the effect of bexarotene on fear memory, and its cognitive effects remain controversial. Therefore, we evaluated the effect of bexarotene on context-dependent and conditioned-stimulus-dependent fear memory.

## Results

**Figure 1:**
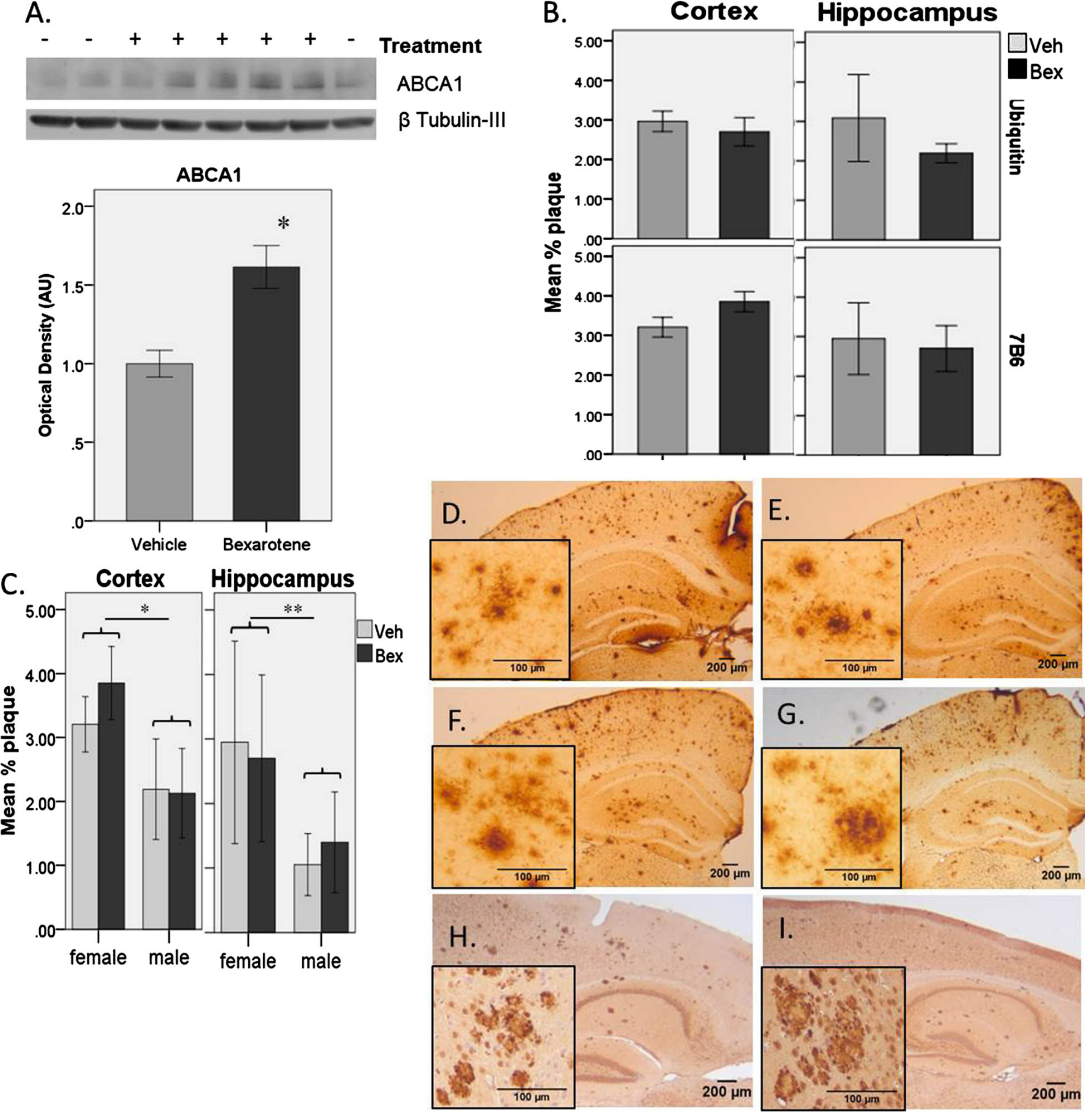
**Bexarotene significantly increases ABCA1 in the brains of *APP***_***swe***_**/*PS1***_***ΔE9***_ **mice, but does not alter Aβ plaques.** (**A**) Representative Western blot (Vehicle (−), Bexarotene (+)) of ABCA1 in cortex of 8.5 mo. female mice, and graph of quantification normalized to β-tubulin-III. Bexarotene significantly increases ABCA1 expression by 50% after three days of gavage treatment [* F(1,13)=5.261, p=0.027]. (**B**) Percent brain area covered by plaques in 8 month (ubiquitin) and 11 month (7B6) female mice, calculated by stereological estimation on sections stained with the antibodies indicated. No significant differences in plaque burden were detected between treatment groups. N-values: Ubiquitin – veh=4, bex=6; 7B6 – veh=3, bex=5. Error bars represent standard deviation. (**C**) Gender comparison between % plaque area in 11 mo. mice, calculated by stereological estimation on sections stained with 7B6 antibody. Males had significantly lower plaque load compared to females in both treatment groups [* cortex, F(1,19)=20.177, p<0.0005 and ** hippocampus F(1,19)=14.045, p=0.001]. Error bars represent standard deviation. N-values: females- veh=3, bex5; males- veh=7, bex=8. Sample size estimates show this experiment has power of 88%. Representative images of plaques in *APP*_*swe*_/*PS1*_*ΔE9*_ mice, stained with 7B6 - (**D**) M Veh (**E**) M Bex (**F**) F Veh (**G**) F Bex - and ubiquitin - (**H**) F Veh (**I**) F Bex.

To confirm the apparent benefit of acute treatment with bexarotene [13], we initially dosed two cohorts of 8 month old female *APP*_*swe*_/*PS1*_*ΔE9*_ mice with 100 mg/kg of this RXR agonist (or indicated vehicle) by daily oral gavage. We analyzed the effects of treatment on ABCA1 levels in *APP*_*swe*_/*PS1*_*ΔE9*_ mice, and found that the use of DMSO or corn oil as a vehicle does not create a significant difference in the relative means of bexarotene treated groups normalized to their respective vehicle [(*M*=1.570, *SD*=0.203) and (*M*=1.641, *SD*=0.157), respectively *F*(1,10)=0.044, *p*=0.837], and that there was no interaction between vehicle used and treatment group. As expected, we found that bexarotene significantly increased levels of ABCA1 by 50% after three days of treatment (Figure 1A) [* *F*(1,13)=5.261, *p*=0.027], confirming that this compound engaged the expected targets in the brains of these mice. To assess the impact of bexarotene on amyloid burden in male and female mice, we dosed an 8 month (DMSO vehicle) and an 11 month old (corn oil vehicle) cohort of *APP*_*swe*_/*PS1*_*ΔE9*_ mice. Again, we found no significant effect of vehicle type on Aβ levels [cortex *F*(1,29)=0.277, *p*=0.965, hippocampus *F*(1,29)=3.266, *p*=0.081], and no interaction between vehicle used and treatment group. In contrast to findings of Cramer *et al*. [13], no significant differences in Aβ plaque burden were observed in brains of bexarotene treated *APP*_*swe*_/*PS1*_*ΔE9*_ mice compared to vehicle [cortex *F*(1,29)=0.002, *p*=0.965, hippocampus *F*(1,29)=0.398, *p*=0.533] (Figure 1B). Though the sample sizes for each group are small, sample size estimates for each experiment showed that they each have a power ≥88%. In addition, the inability of bexarotene to alter plaque levels in this model has recently been reported by additional groups [14-17]. While there has been increasing evidence that plaques may not be as informative correlates of disease as soluble forms of Aβ, the inability of bexarotene to alter soluble Aβ levels in this mouse model has also been recently reported [14-17]. Notably, we observed a significant main effect of gender on plaque levels in 11 month old mice. Male groups had significantly lower plaque load compared to female groups [* cortex, *F*(1,19)=20.177, *p*<0.0005 and ** hippocampus *F*(1,19)=14.045, *p*=0.001] (Figure 1C). In 8 month old mice, males were tested, so that age group could not be included in the analysis for a gender effect, but gender differences in plaque load have been reported as early as six months of age in this mouse model [18].

**Figure 2:**
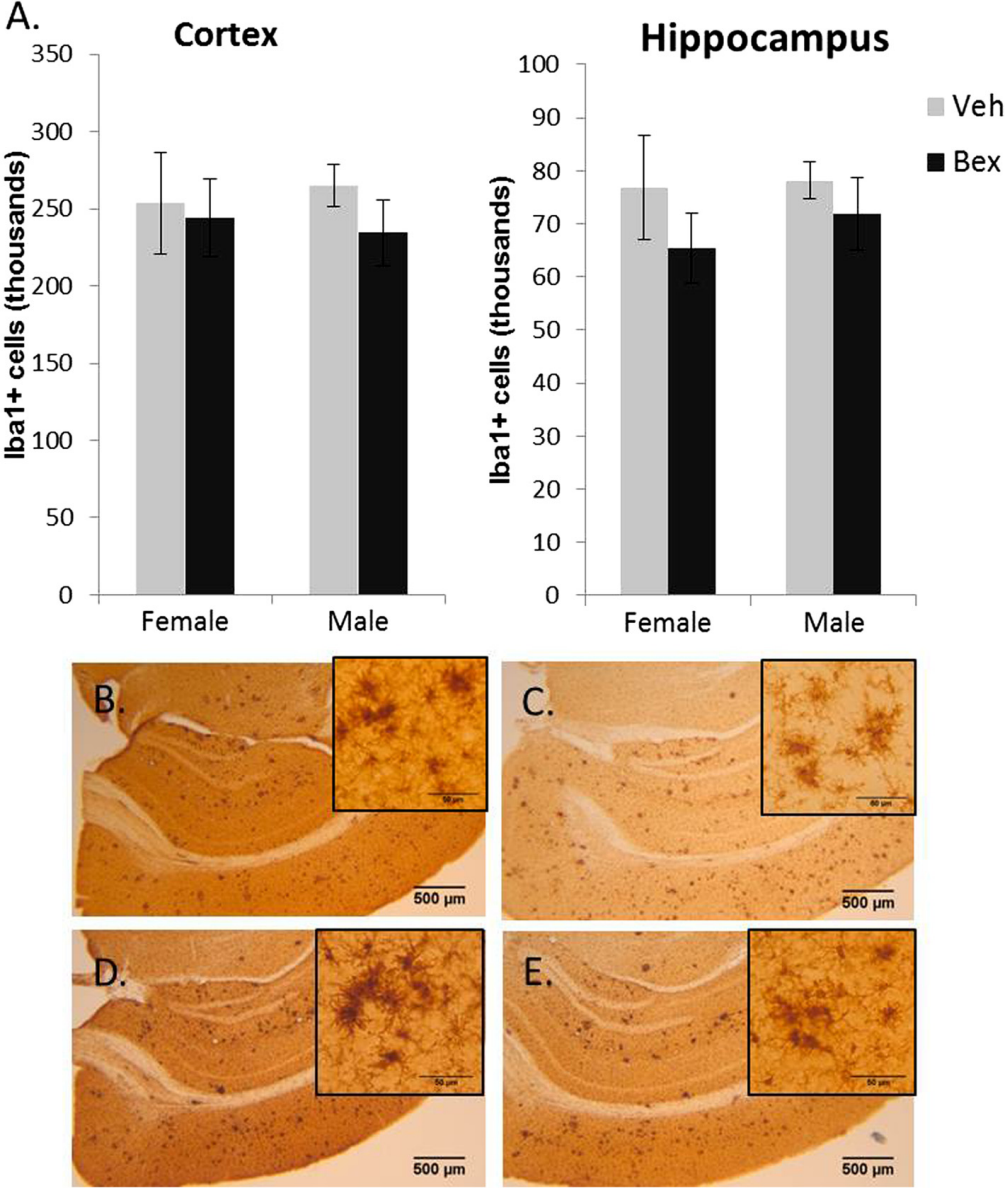
**Immunoreactivity of glia is unchanged in *APP***_***swe***_**/*PS1***_***ΔE9***_ **mice following administration of bexarotene.** (**A**) IBA1 levels in 11 month old male and female *APP*_*swe*_/*PS1*_*ΔE9*_ mice are unaffected by bexarotene treatment in both cortex [*F*(1,27)=2.231, *p*=0.147] and hippocampus [*F*(1,27)=2.127, *p*=0.156], and no significant effect of gender was detected [*F*(1,27)=0.665, *p*=0.422]. No significant interactions were detected. Error bars represent standard deviation. N-values: non-transgenics (veh), males=5, females=5; *APP*_*swe*_/*PS1*_*ΔE9*,_ males: Bex =8, Veh=7, females: Bex=5, Veh=3. Representative images of IBA1 staining and microglial morphology in (**B**) F tg Veh, (**C**) F tg Bex, (**D**) M Tg Veh and (**E**) M Tg Bex. Scale bars = 500 um large images and 50 um on inserts. Morphology shows an amoeboid (active) state with clusters of activated microglia in *APP*_*swe*_/*PS1*_*ΔE9*_ animals (**B**,**D**). This morphology and clustering appears unchanged by treatment with bexarotene (**C**,**E**).

Analysis of microglial immunoreactivity using an antisera specific to ionized calcium binding adaptor molecule 1 (Iba1), a microglial inflammatory and phagocytic marker [22], revealed a similar pattern between *APP*_*swe*_/*PS1*_*ΔE9*_ mice treated with bexarotene or vehicle (Figure 2). Despite the potential of RXR agonists to modulate the microglial activation present in *APP*_*swe*_/*PS1*_*ΔE9*_ mice, no significant effect of bexarotene was observed compared with vehicle administration [cortex *F*(1,27)=2.231, *p*=0.147, and hippocampus *F*(1,27)=2.127, *p*=0.156] (Figure 2A). No significant main effect of gender was found, and no interaction was detected between gender and treatment. Microglial morphology was also analyzed, as described in [23]. Microglia in transgenic female (Figure 2B) and male (Figure 2D) animals show a transitional amoeboid (activating) morphology with clustering of these activated microglia, which was unchanged after administration of bexarotene (Figure 2C and E, respectively). In contrast, microglia from female and male non-transgenic animals have a uniformly ramified, resting morphology as shown in Additional File 1. A recent study analyzing the presence of Aβ in Iba1-positive microglia also failed to show an effect of bexarotene on Aβ uptake and clearance [17].

**Figure 3:**
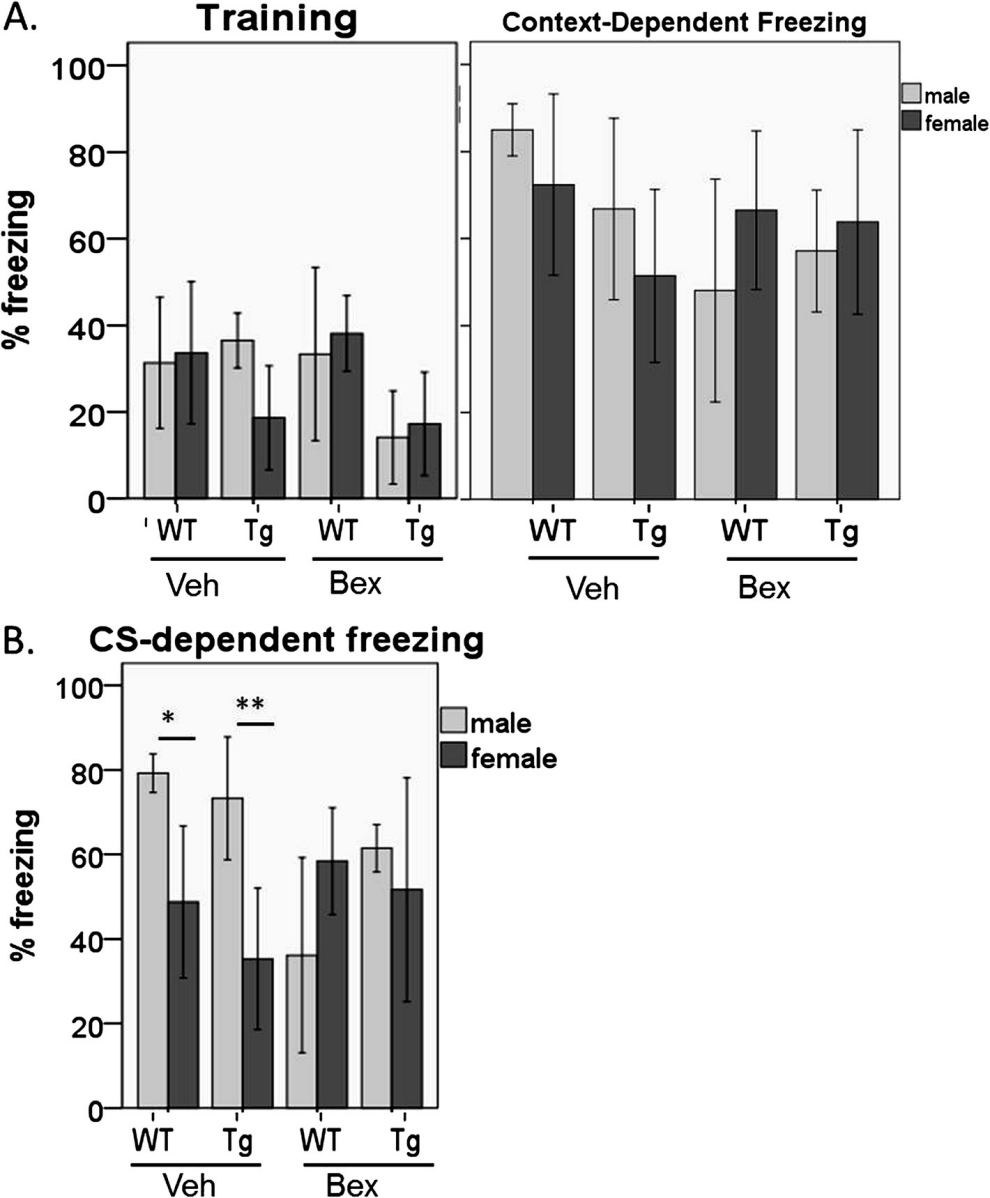
**Bexarotene fails to produce an effect on context-dependent or CS-dependent freezing behavior in *APP***_***swe***_***/PS1***_***ΔE9***_ **mice.** Percent of time spent freezing was measured to quantify long term fear memory. (**A**) In the training session, *APP*_*swe*_/*PS1*_*ΔE9*_ mice receiving bexarotene treatment demonstrated similar levels of context-dependent freezing compared to their vehicle-treated counterparts [*F*(7,44)=3.905, *p*=0.002; Tukey: male nontg *p*=1.000, male tg *p*=0.182, female nontg *p*=0.999, female tg *p*=1.000]. In the context-dependent testing session, no significant differences were detected among any of the groups [*F*(7,44)=1.794, *p*=0.113]. (**B**) Analysis of CS-dependent fear responses show no significant effect of bexarotene on freezing behavior in *APP*_*swe*_/*PS1*_*ΔE9*_ male or female mice. A significant gender difference was detected between males and females in both non-transgenic and *APP*_*swe*_/*PS1*_*ΔE9*_ animals treated with vehicle [main effect of gender *F*(1,15)=23.506, *p*<0.0005; main effect of genotype *F*(1,15)=1.898, *p*=0.189, no significant interaction; females [M=42.006,SD=17.65] show lower levels of freezing than males [M=76.260,SD=10.50]. These gender-related differences were not observed in mice treated with bexarotene [males M=48.79, SD=20.71; females M=54.55, SD=21.48; main effect of sex *F*(1,27)=0.630, *p*=0.434; main effect of genotype *F*(1,27)=1.388,*p*=0.249; no significant interaction]. The condition of equality of variances was not met for this test. For all parts, error bars represent standard deviation. N-values females: nontg-veh=4, AP-veh=7, nontg-bex=8, AP-bex=13. N-values males: nontg-veh=4, AP-veh=4, nontg-bex=6, AP-bex=5.

Finally, we analyzed the effects of bexarotene treatment on context-dependent freezing behavior in 8 month old female *APP*_*swe*_/*PS1*_*ΔE9*_ and littermate non-transgenic mice according to the protocol described by Cramer *et al*. [13]. Behavioral measures in males and females were assessed separately due to gender differences present in this mouse model [18]. In the training session, *APP*_*swe*_/*PS1*_*ΔE9*_ mice receiving vehicle and bexarotene treatment demonstrated similar levels of context-dependent freezing [*F*(7,44)=3.905, *p*=0.002, Tukey: male nontg *p*=1.000, male tg *p*=0.182, female nontg *p*=0.999, female tg *p*=1.000] (see Figure 3A) indicating that acquisition of fear response was not significantly affected by genotype, sex, or treatment.

**Figure 4:**
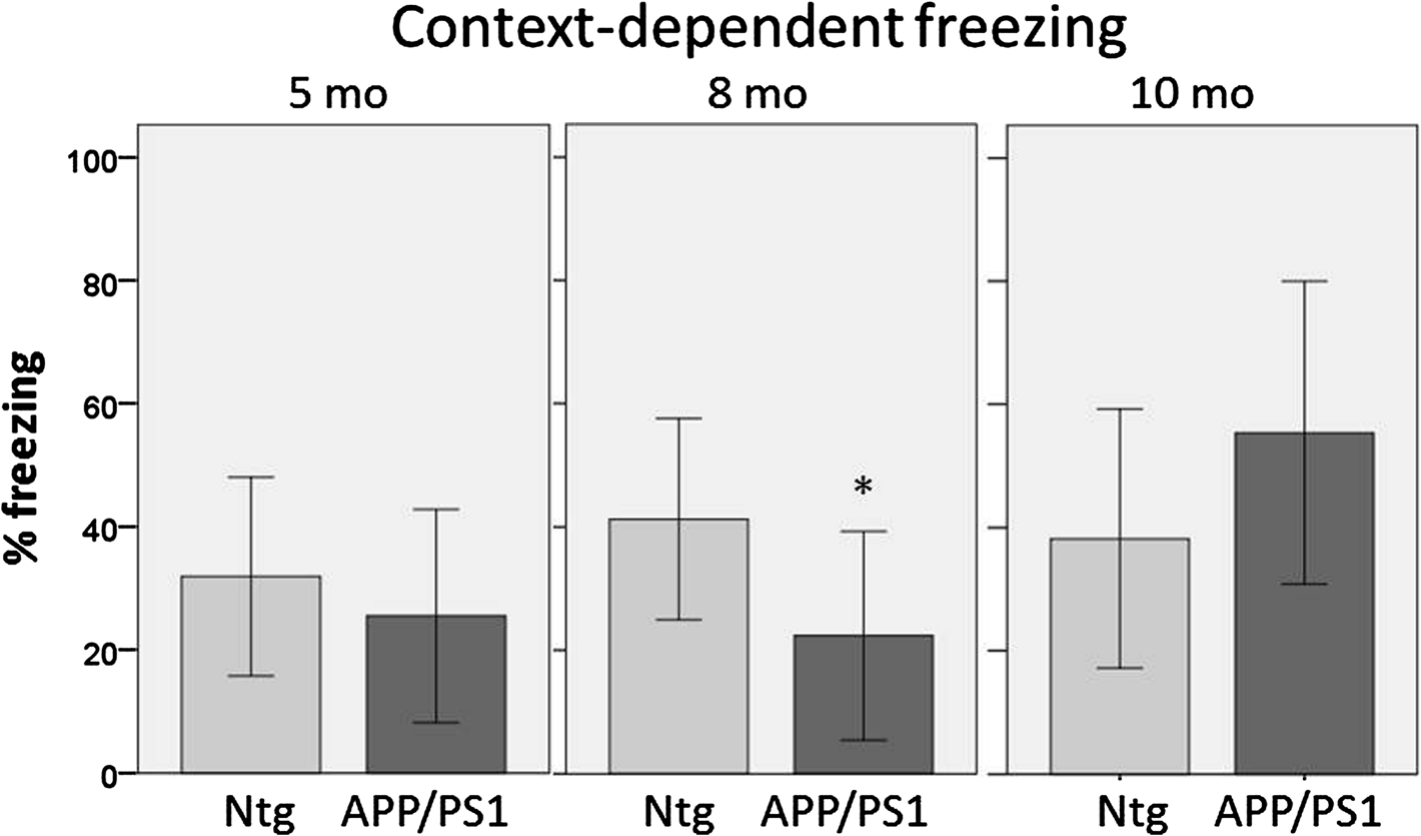
**Context-dependent freezing in *APP***_***swe***_***/PS1***_***ΔE9***_ **female mice and non-transgenic littermates following a high-sensitivity conditioning protocol.** A deficit is not yet present in *APP*_*swe*_/*PS1*_*ΔE9*_ females at 5 months of age [*F*(1,17)=0.684, *p*=0.420]. Context-dependent freezing deficits are detectable in *APP*_*swe*_/*PS1*_*ΔE9*_ females at 8 months of age [*F(1,17)=5.943, *p*=0.026], but do not persist to 10 months of age [*F*(1,18)=2.824, *p*=0.110]. N-values: 5 month- nontg=9, APP/PS1=10, 8 month- nontg=8, APP/PS1=11, 10 month- nontg=12, APP/PS1=8. Error bars represent standard deviation.

Testing for context-dependent fear memory yielded no significant differences among any of the groups [*F*(7,44)=1.794, *p*=0.113]. This finding was not surprising given the strong unconditioned stimulus and its multiple presentations in the protocol described in [13], which was used here for replication purposes. Of particular note, the group means for context-dependent freezing behavior in Figure 3A were almost double those reported in Cramer et al. [13] using these testing parameters. We also performed a high-sensitivity alternative conditioning paradigm, consisting of a short pre-exposure period and a single delivery of the unconditioned stimulus as described in [24] and the Methods. Using this paradigm we detected a significant freezing deficit in 8 month old *APP*_*swe*_/*PS1*_*ΔE9*_ females compared to non-transgenic littermates [*F*(1,17)=5.943, *p*=0.026] (Figure 4). However, this deficit was not persistent at 10 months of age [*F*(1,18)=2.824, *p*=0.110] with an opposing trend of increased freezing in *APP*_*swe*_/*PS1*_*ΔE9*_ females (Figure 4). These data indicate that freezing behavior might not be robust or reliable measure of cognitive deficits in this mouse model.

We also analyzed conditioned stimulus (CS)-dependent fear responses, and found no effect of bexarotene on freezing behavior in *APP*_*swe*_/*PS1*_*ΔE9*_ mice compared to non-transgenic littermates (Figure 3B). However, we again observed an unexplained trend of freezing deficit in non-transgenic males treated with bexarotene as compared to vehicle- and bexarotene-treated *APP*_*swe*_/*PS1*_*ΔE9*_ males (Figure 3B). In addition, a significant gender difference was also detected in this task between males and females of vehicle-treated non-transgenic and *APP*_*swe*_/*PS1*_*ΔE9*_ animals [main effect of gender *F*(1,15)=23.506, *p*<0.0005; main effect of genotype *F*(1,15)=1.898, *p*=0.189, no significant interaction] with females [M=42.006,SD=17.65] showing lower levels of freezing than males [M=76.260,SD=10.50] (Figure 3B). These gender-related differences were no longer observed in mice treated with bexarotene [males M=48.79, SD=20.71, females M=54.55, SD=21.48; main effect of sex *F*(1,27)=0.630, *p*=0.434; main effect of genotype *F*(1,27)=1.388,*p*=0.249; no significant interaction. The condition of equality of variances was not met for this test].

## Discussion

Our data failed to replicate the apparent benefit of acute bexarotene treatment in mouse models of Aβ amyloidosis previously reported [13]. It is unknown whether chronic administration of bexarotene or alternate dosing strategies would have a beneficial effect. Nevertheless, our failure to replicate the initial observations emphasizes the importance of gender-related differences in the *APP*_*swe*_/*PS1*_*ΔE9*_ mouse model, and other work indicates that these differences may extend to humans [19,20]. Since the completion of this study, bexarotene has been tested in other animal models of Aβ amyloidosis, including APP/PS1-21 [17], 5XFAD [17], and *APP*_*swe*_/*PS1*_*ΔE9*_ with APOE3/4 allele knock-in [14], as summarized in Table 1. All have failed to replicate a change in soluble or plaque forms of Aβ, with the exception of the non-pathogenic soluble Aβ40 species, which decreased after acute treatment with bexarotene in the 5XFAD mouse model, but not in the *APP*_*swe*_/*PS1*_*ΔE9*_ or the APP/PS1-21 models [17].

**Table 1:** Summary of recently published findings on bexarotene in mouse models of Aβ amyloidosis related to the study presented here (first row)

**Model**	**Age (months)**	**Days of treatment**	**Brain insoluble/plaque Aβ**	**Brain soluble Aβ**	**Fear conditioning**
*APP*_swe_/*PS1*_∆E9_	8 and 11	3, 7 and 14,	No effect	N/A	No effect
APP/PS1 + APOE3/4 [14]	7	15	No effect	No effect	N/A
APP _swe_/PS1_∆E9_[15]	7	11	No effect	No effect	N/A
APP _swe_/PS1_∆E9_[16]	10	14	No effect	No effect	N/A
*APP*_swe_/*PS*1_∆E9_[17]	6	7	No effect	No effect	N/A
5XFAD[17]	3-4	7	No effect	Decrease Aβ40 only	N/A
APP/PS1-21[17]	9	26	No effect	No effect	N/A

N/A indicates that the listed data point was not reported. Superscript numbers indicate reference number of the original work.

Importantly, we are unable to replicate a robust and persistent fear conditioning deficit in the *APP*_*swe*_/*PS1*_*ΔE9*_ mouse model, even with an alternate, highly sensitive testing paradigm [24]. Fear conditioning is the sole behavioral measure previously used to test bexarotene in this model [13]. In *APP*_*swe*_/*PS1*_*ΔE9*_ mice with APOE3/4 knock-in, bexarotene was reported to rescue deficits in the Radial Arm Water Maze at 7 months of age [14], but this testing was also performed on mixed gender cohorts of n=5 per group, introducing a potential false-positive treatment effect. Therefore, the current cognitive preclinical data do not validate the potential of this drug for Alzheimer’s therapy.

Together, the array of published work on the effect of bexarotene on pathology and cognitive impairment in mouse models of Aβ amyloidosis produces no rigorous evidence that bexarotene is a suitable candidate for the treatment of Alzheimer’s disease (Table 1). There are potential discrepancies in the formulation of bexarotene used in replicating studies as compared to the original experiments [13,16] due to lack of availability of this formulation to other research groups. While the consistent upregulation of ABCA1 and ApoE using various formulations of bexarotene [13-17] indicates that it does indeed activate the proposed targets, these may not be the true mechanism of action responsible for the changes observed in Cramer *et al*. [13]. In light of our and other groups [14-17] overall failures to replicate the key findings on bexarotene in mouse models of Aβ amyloidosis, we recommend that the original formulation be made available for rigorous preclinical studies in multiple mouse models in order to clarify the existing discrepancies and to elucidate the mechanism of action before clinical testing progresses.

## Methods

### Animals

*APP*_*swe*_/*PS1*_*ΔE9*_ (B6.Cg-Tg(APPswe,PSEN1dE9)85Dbo/Mmjax) were used. 8 month and 11 month old *APPswe*/*PS1ΔE9* mice were housed on a 14 hr dark:10 hr light cycle in standard cages with 2–5 mice each and given free access to food and water. Bexarotene (ChemieTek) was dissolved in a vehicle of <15% DMSO in milk or in corn oil, as indicated in the corresponding figure legends. Mice were weighed at the beginning of the treatment period and the necessary volume of solubilized bexarotene was calculated to produce a concentration of 100 mg/kg for each animal. Animals were gavaged with vehicle or bexarotene solution daily for 3, 7, or 14 days, as indicated. Studies were performed on mixed gender or females only, where indicated.

### Brain ABCA1 quantification

Mice were sacrificed two hours after the day three gavage using brief CO_2_ exposure, followed immediately by decapitation. Brains were removed and one hemisphere was collected for ABCA1 quantification. Cortices were quickly dissected on ice with cold forceps. Samples were placed on dry ice in labeled, pre-cooled microfuge tubes. Samples were frozen at −80°C until use. Fresh, cold RIPA buffer (10× volume of sample) with Protease Inhibitor Cocktail was added to samples on ice and tissue was mechanically dissociated using 1mL pipet tips. Samples were sonicated with brief pulses on ice, and centrifuged at 14000 rpm for 25 min at 4°C. Supernatants were transferred to new labeled microfuge tubes and kept on ice during protein estimation, or stored at −80°C until use. Protein concentration was estimated using the BCA Assay, and analyzed by the Epoch Com6 spectrophotometer (BioTek) with Gen5 analysis software. Samples for protein blot were prepared containing 1.0-1.5 μg protein/μL of sample solution. Samples were mixed, heated at 70°C for five minutes, then cooled and a volume containing 30 μg of protein was loaded into wells of 4-12% Bis-Tris gels (Novex) with MOPS running buffer. Gel was run for 120 min at 120 volts, then transferred to a PVDF membrane for 150 min at 30v. Membranes were probed with ABCA1 mouse monoclonal antibody and with antisera against β-tubulin III as a loading control, and were incubated with HRP-conjugated secondary antibodies and exposed to HRP substrate. Exposed films were scanned without any background- or noise-subtraction and analyzed using ImageJ software. The relative optical density of each band was calculated using the area under the curve of lane profile plots on unaltered images. The experiment was replicated in triplicate, and the mean optical density (OD) was calculated.

### Aβ Plaque quantification

Mice were gavaged with 100 mg/kg bexarotene for 14 days and sacrificed as described above, and hemispheres were immediately immersed in 4% paraformaldehyde for 24 hours at 4°C. Brains were then moved to PBS solution for 24 hours at 4°C, followed by another change of PBS for storage until use. The tissue was then embedded in paraffin and sectioned into 10 μm coronal slices (7B6-stained) or 10 μm sagittal slices between 1.2 and 1.5 mm from the midline (ubiquitin-stained). Sections were mounted onto slides and stained with ubiquitin (Dako) or 7B6 antibodies (Abcam) to detect Aβ plaques. Plaque area was calculated using the Area Fraction Fractionator probe in the Stereo Investigator stereology program. The counting frame was 150x150 with gridsizes 500x500 μm for cortex and 300x300 μm for hippocampus, and a grid spacing of 10 μm. Plaque area for hippocampus and cortex were measured and normalized to the total area of the brain region for each animal. Data was collected and verified by two independent investigators, blinded to groups.

### Iba-1 Quantification and morphological analysis

Animals were sacrificed after 14 days of gavage and their tissues perfused. The brain tissue of each of the animals was removed and placed in a 30% sucrose preservative, and sectioned to a thickness of 40 μm on a freezing microtome. Every eighth section was immunostained with anti-Iba1 antibody (1:1000, Wako, cat. #019-19741). Morphology of resting (ramified) and active (amoeboid) microglia was analyzed according to the guidelines specified in [23]. Briefly, ramified microglia are identified with a small cell body and long, thin projections with distal branching. In contrast, active microglia exhibit a shortening of projections and enlargement of the cell body as they are reabsorbed, leading to an amoeboid morphology.

### Fear conditioning

All mice used for these studies were generated by breeding a single cohort of 10 male and 20 female mice from the line described above. Animals were gavaged with vehicle or bexarotene (100 mg/kg) solution daily for 7 days. Behavioral tests were performed in the morning before the daily gavage to minimize any effect of stress from this procedure on behavior. Three days before the start of the test period, animals were handled and the tails marked with permanent marker to eliminate stress caused by checking ear-tags just prior to behavioral testing. The following two days before behavioral testing, each animal was handled and separately habituated to empty waiting cages in a separate room adjacent to the testing room for ten minutes.

#### Protocol 1

Context- and Cue-dependent fear conditioning was conducted as described in Cramer et al. [13], beginning after 4 days of gavage (before the 5th administration). Conditioning was performed in a test chamber with a shock grid floor and a contextual striped and checkered pattern on three of the walls. Testing and data collection were automated by ANY-Maze 4.70 software (Stoelting Co., Wood Dale, IL) according to the following protocol parameters: Light in the chamber 1.5 visible + 1.5 Infrared, Fan ON (65 dB), smell 30% Ethanol, Freezing ON Threshold = 25 msec, Freezing OFF Threshold = 30 msec, Minimum Freezing Duration = 500 msec.

#### Training session

Mice were placed in empty waiting cages for ten minutes prior to the start of the test, then placed in the test chamber and allowed to explore for 120 seconds. Mice were then exposed to a conditioned stimulus (CS, 83dB and 2800Hz) for 30 seconds, followed by a 2 second delay, and finally the unconditioned stimulus (US, 0.6 mA) for two seconds. Mice remained in the chamber for an additional 30 seconds to measure immediate freezing response following the CS/US pairing. This sequence was repeated four times for each animal.

#### Context dependent freezing

Context-dependent fear behavior was analyzed 24 hours later. Mice were placed in the same test chamber for five minutes without any CS or US administration, and freezing behavior was measured throughout the test.

#### Conditioned Stimulus (CS)-dependent freezing

An additional 24 hours later, mice were tested for CS-dependent freezing behavior. Mice were placed in a novel test chamber with plain white walls on all sides and a solid floor. Bedding was placed in the bottom of this new chamber, as well as a small paper towel with 1:100 dilution of vanilla extract (McCormick) in water. Mice were allowed to explore the chamber for 120 seconds, followed by CS presentation for 30 seconds, and a 30 second delay to measure immediate freezing response. This sequence was repeated four times for each animal.

#### Protocol 2

A high-sensitivity context-dependent fear conditioning protocol (modified from *24*) was also tested in separate cohorts of *APP*_*swe*_/*PS1*_*ΔE9*_ female mice at 5, 8 and 10 months of age. Mice were habituated in the same manner as described previously. The test chamber was identical, with the addition of a scent cue (coconut extract 1:500 in water, McCormick) placed underneath the grid floor. In the Training Session, mice were placed in the test chamber and allowed to explore for 30 seconds. Mice were then exposed to the same unconditioned stimulus as in the previous paradigm (US, 0.6 mA) for two seconds. Mice remained in the chamber for an additional 35 seconds to measure immediate freezing response. 24 hours later, mice were placed in the same test chamber for four minutes without US administration, and time freezing was measured.

### Statistical analysis

Data were analyzed with Univariate Analysis of Variance (ANOVA) using SPSS Statistics 20 software. For Figure 1, analysis was performed on data in 2x2 (treatment x vehicle or treatment x sex) between-subjects factorial design. For Figures 2, 3 and 4, analysis was performed in a 2x2 (genotype x sex) between-subjects factorial design for each treatment group, or a 2x2x2 (genotype x sex x treatment) between-subjects factorial design, and main effects and interactions were analyzed. Homogeneity of variance was met for all tests, unless noted otherwise. Significant *p*-value was set to 0.05.

## Ethical approval

All research was performed under the guidelines of the Animal Care and Use Committee of Johns Hopkins University under protocol M012M183, and following established OSHA guidelines.

## Competing interests

The author(s) declare that they have no competing interests.

## Authors’ contributions

KL and KM performed the primary work on these experiments and wrote the manuscript text. KL prepared the figures. DL assisted in animal gavage and JA in assisted in performing animal surgery, dissections and tissue preparation. AS provided the interpretation of the cognitive testing protocol reported in the original study and prepared all necessary materials and software templates for cognitive testing. AS, JT, and PW provided guidance throughout the project and edited the manuscript. All authors read and approved the final manuscript.

## Supplementary Material

Additional file 1 **Representative images of IBA1 stained microglia from female (A) and male (B) non-transgenic animals.** Microglia have a uniformly ramified, resting morphology. Scale bars = 500 um in wide field images and 50 um on inserts. Click here for file

## References

[B1] Jankowsky JL Fadale DJ Anderson J Xu GM Gonzales V Jenkins NA Copeland NG Lee MK Younkin LH Wagner SL Younkin SG Borchelt DR Mutant presenilins specifically elevate the levels of the 42 residue beta-amyloid peptide in vivo: evidence for augmentation of a 42-specific gamma secretase Hum Mol Genet 2004 13 2 159 70 14645205 10.1093/hmg/ddh019

[B2] Burgess BL McIsaac SA Naus KE Chan JY Tansley GH Yang J Miao F Ross CJ van Eck M Hayden MR van Nostrand W St George-Hyslop P Westaway D Wellington CL Elevated plasma triglyceride levels precede amyloid deposition in Alzheimer's disease mouse models with abundant a beta in plasma Neurobiol 2006 24 1 114 27 10.1016/j.nbd.2006.06.007 16899370

[B3] Wong PC Savonenko A Li T Price DL In Basic neurochemistry ***Neurobiology of Alzheimer’s disease*** 2012 Oxford: Elsevier Inc: Eighth Edition. Edited by Brady S, Albers P 815 828

[B4] Jiang Q Lee CY Mandrekar S Wilkinson B Cramer P Zelcer N Mann K Lamb B Willson TM Collins JL Richardson JC Smith JD Comery TA Riddell D Holtzman DM Tontonoz P Landreth GE ApoE promotes the proteolytic degradation of abeta Neuron 2008 58 5 681 93 10.1016/j.neuron.2008.04.010 18549781 PMC2493297

[B5] Hashimoto T Serrano-Pozo A Hori Y Adams KW Takeda S Banerji AO Mitani A Joyner D Thyssen DH Bacskai BJ Frosch MP Spires-Jones TL Finn MB Holtzman DM Hyman BT Apolipoprotein E, especially apolipoprotein E4, increases the oligomerization of amyloid β peptide J Neurosci 2012 32 43 15181 92 10.1523/JNEUROSCI.1542-12.2012 23100439 PMC3493562

[B6] Roses AD Saunders AM APOE is a major susceptibility gene for Alzheimer's disease Curr Opin Biotechnol 1994 5 6 663 667 10.1016/0958-1669(94)90091-4 7765750

[B7] Castellano JM Kim J Stewart FR Jiang H DeMattos RB Patterson BW Fagan AM Morris JC Mawuenyega KG Cruchaga C Goate AM Bales KR Paul SM Bateman RJ Holtzman DM Human ApoE isoforms differentially regulate brain amyloid-β peptide clearance Sci. Transl. Med 2011 3 89 89ra57 10.1126/scitranslmed.3002156 21715678 PMC3192364

[B8] Tokuda T Calero M Matsubara E Vidal R Kumar A Permanne B Zlokovic B Smith JD LaDu MJ Rostagno A Frangione B Ghiso J Lipidation of apolipoprotein E influences its isoform-specific interaction with Alzheimer's amyloid beta peptides Biochem J 2000 348 359 65 10.1042/0264-6021:3480359 10816430 PMC1221074

[B9] Hirsch-Reinshagen V Zhou S Burgess BL Bernier L McIsaac SA Chan JY Tansley GH Cohn JS Hayden MR Wellington CL Deficiency of ABCA1 impairs apolipoprotein E metabolism in brain J Biol Chem 2004 279 41197 41207 10.1074/jbc.M407962200 15269218

[B10] Wahrle SE Jiang H Parsadanian M Legleiter J Han X Fryer JD Kowalewski T Holtzman DM ABCA1 is required for normal central nervous system ApoE levels and for lipidation of astrocyte-secreted apoE J Biol Chem 2004 279 40987 40993 10.1074/jbc.M407963200 15269217

[B11] Bell RD Sagare AP Friedman AE Bedi GS Holtzman DM Deane R Zlokovic BV Transport pathways for clearance of human Alzheimer's amyloid beta-peptide and apolipoproteins E and J in the mouse central nervous system J Cereb Blood Flow Metab 2007 27 909 918 17077814 10.1038/sj.jcbfm.9600419 PMC2853021

[B12] Morikawa M Fryer JD Sullivan PM Christopher EA Wahrle SE DeMattos RB O'Dell MA Fagan AM Lashuel HA Walz T Asai K Holtzman DM Production and characterization of astrocyte-derived human apolipoprotein E isoforms from immortalized astrocytes and their interactions with amyloid-beta Neurobiol Dis 2005 19 66 76 10.1016/j.nbd.2004.11.005 15837562

[B13] Cramer P Cirroto J Wesson D Lee CY Karlo J Zinn A Casali B Restivo J Goebel W James M Brunden K Wilson D Landreth GE ApoE-directed therapeutics rapidly clear β-amyloid and reverse deficits in AD mouse models Science 2012 23 335 1503 1506 22323736 10.1126/science.1217697 PMC3651582

[B14] Fitz NF Cronican AA Lefterov I Koldamova R Comment on “ApoE-directed therapeutics rapidly clear beta-amyloid and reverse deficits in AD mouse models Science Tech. Comments 2013 340 924-c 10.1126/science.1235809 PMC4086452 23704552

[B15] Price AR Xu G Siemienski ZB Smithson LA Borchelt DR Golde TE Felsenstein KM Comment on “ApoE-directed therapeutics rapidly clear beta-amyloid and reverse deficits in AD mouse models Science Tech. Comments 2013 340 924-d 10.1126/science.1234089 23704553

[B16] Tesseur I Lo AC Roberfroid A Dietvorst S Van Broeck B Borgers M Gijsen H Moechars D Mercken M Kemp J D’Hooge R De Strooper B Comment on “ApoE-directed therapeutics rapidly clear beta-amyloid and reverse deficits in AD mouse models” Science Tech. Comments 2013 340 924-e 10.1126/science.1233937 23704554

[B17] Veeraraghavalu K Zhang C Miller S Hefendehl JK Rajapaksha TW Ulrich J Jucker M Holtzman DM Tanzi RE Vassar R Sisodia SS Science Tech. Comments 2013 340 924-f 10.1126/science.1235505 23704555

[B18] Callahan MJ Lipinski WJ Bian F Durham RA Pack A Walker LC Augmented senile plaque load in aged female β-amyloid precursor protein – transgenic mice Am 2001 158 3 1173 1177 10.1016/S0002-9440(10)64064-3 11238065 PMC1850367

[B19] Kim J Castellano JM Jiang H Basak JM Parsadanian M Pham V Mason SM Paul SM Holtzman DM Overexpression of low density lipoprotein receptor in the brain markedly inhibits amyloid deposition and increases extracellular a-beta clearance Neuron 2009 64 632 644 10.1016/j.neuron.2009.11.013 20005821 PMC2787195

[B20] Damoiseaux JS Seeley WW Zhou J Shirer WR Coppola G Karydas A Rosen HJ Miller BL Kramer JH Greicius MD Gender modulates the APOE ϵ4 effect in healthy older adults: convergent evidence from functional brain connectivity and spinal fluid tau levels J Neurosci 2012 32 24 8254 62 10.1523/JNEUROSCI.0305-12.2012 22699906 PMC3394933

[B21] Zelcer N Khanlou N Clare R Jiang W Reed-Geaghan EG Landreth GE Vinters HV Tontonoz P Attenuation of neuroinflammation and Alzheimer’s disease pathology by liver x receptors Proc Natl Acad Sci USA 2007 104 25 10601 6 10.1073/pnas.0701096104 17563384 PMC1890560

[B22] Ohsawa K Imai Y Kanazawa H Sasaki Y Kohsaka S Involvement of Iba1 in membrane ruffling and phagocytosis of macrophages/microglia J 2000 113 3073 3084 10934045 10.1242/jcs.113.17.3073

[B23] Kettenmann H Hanisch U-K Noda M Verkhratsky A Physiology of microglia Physiol 2011 91 2 461 553 10.1152/physrev.00011.2010 21527731

[B24] McHugh TJ Tonegawa S CA3 NMDA receptors are required for the rapid formation of a salient contextual representation Hippocampus 2009 19 1153 1158 10.1002/hipo.20684 19650121 PMC2788054

