# Correction: treatment with bexarotene, a compound that increases apolipoprotein-E, provides no cognitive benefit in mutant APP/PS1 mice

**DOI:** 10.1186/1750-1326-8-26

**Published:** 2013-08-08

**Authors:** Katherine D LaClair, Kebreten F Manaye, Dexter L Lee, Joanne S Allard, Alena V Savonenko, Juan C Troncoso, Philip C Wong

**Affiliations:** 1 Department of Pathology, The Johns Hopkins School of Medicine, 720 Rutland Avenue, Ross 558, Baltimore, MD 21205, USA; 2 Department of Neuroscience, The Johns Hopkins School of Medicine, 720 Rutland Avenue, Ross 558, Baltimore, MD 21205, USA; 3 Department of Neurology, The Johns Hopkins School of Medicine, 720 Rutland Avenue, Ross 558, Baltimore, MD 21205, USA; 4 Program in Cellular and Molecular Medicine, The Johns Hopkins School of Medicine, 720 Rutland Avenue, Ross 558, Baltimore, MD 21205, USA; 5 Department of Physiology and Biophysics, Howard University College of Medicine, 520 W Street, NW Suite 2305, Washington, DC 20059, USA

## 

Correction to LaClair et al.: Treatment with bexarotene, a compound that increases apolipoprotein-E, provides no cognitive benefit in mutant APP/PS1 mice. Molecular Neurodegeneration 2013 8:18.

After publication of this work [1], we were alerted to an error in the reported cohort size used for the Radial Arm Water Maze as referenced from [2]. The correct cohort size reported in [2] is n=8-13 for each group of mixed gender animals. In addition, it came to our attention that our analysis of a potential false-positive treatment effect caused by gender differences, and therefore bexarotene’s inability to rescue cognitive decline, may appear to be contradicted by the statement that it “was equally effective in both genders” of transgenic mice [2]. However, direct experimental data supporting this statement is not presented. In addition, this statement does not address any differences in the performance of each gender, only that bexarotene appears effective in each gender separately, and the statistical significance of this reported effect remains unknown. Therefore, we maintain that the currently published cognitive preclinical data, including our own, do not validate the potential of bexarotene for Alzheimer’s therapy.”
