# Supplementary figures and images for: Treatment with bexarotene, a compound that increases apolipoprotein-E, provides no cognitive benefit in mutant APP/PS1 mice
Source: Mol Neurodegener. 2013 Jun 13;8:18. doi: 10.1186/1750-1326-8-18 (PMC3693923; doi:10.1186/1750-1326-8-18)

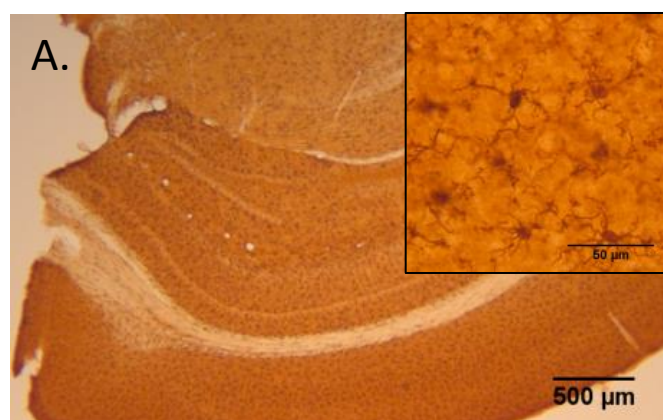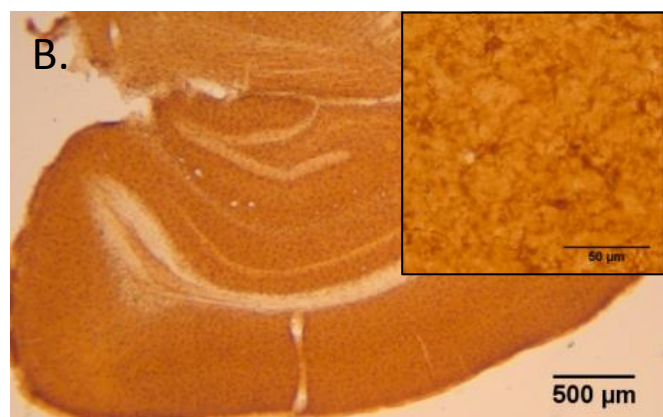

Supplement: Additional file 1 — Representative images of IBA1 stained microglia from female (A) and male (B) non-transgenic animals. Microglia have a uniformly ramified, resting morphology. Scale bars = 500 um in wide field images and 50 um on inserts. [file 1750-1326-8-18-S1.pdf]
